# Recent Advances in Transcatheter Aortic Valve Implantation: Novel Devices and Potential Shortcomings

**DOI:** 10.2174/1573403X09666131202124807

**Published:** 2013-11

**Authors:** J. Blumenstein, C. Liebetrau, A. Van Linden, H. Moellmann, T. Walther, J. Kempfert

**Affiliations:** 1 Kerckhoff-Klinik Heartcenter, Department of Cardiac Surgery, Bad Nauheim, Germany;; 2 Kerckhoff-Klinik Heartcenter, Department of Cardiology, Bad Nauheim, Germany

**Keywords:** Transapical, transfemoral, transcatheter aortic valve implantation, aortic valve stenosis.

## Abstract

During the past years transcatheter aortic valve implantation (TAVI) has evolved to a standard technique for the treatment of high risk patients suffering from severe aortic stenosis. Worldwide the number of TAVI procedures is increasing exponentially. In this context both the transapical antegrade (TA) and the transfemoral retrograde (TF) approach are predominantly used and can be considered as safe and reproducible access sites for TAVI interventions. As a new technology TAVI is in a constant progress regarding the development of new devices. While in the first years only the Edwards SAPIEN™ and the Medtronic CoreValve™ prostheses were commercial available, recently additional devices obtained CE-mark approval and others have entered initial clinical trials. In addition to enhance the treatment options in general, the main driving factor to further develop new device iterations is to solve the drawbacks of the current TAVI systems: paravalvular leaks, occurrence of AV-blocks and the lack of full repositionability.

## INTRODUCTION

Since the first in man implantation back in 2002 (Cribier), the further evolution of transcatheter (T) aortic valve implantations (AVI) has been impressive with numbers of implantations increasing exponentially. While in the first years TAVI was limited to specialized centers only, TAVI in Europe today can be considered a routine intervention. As such in 2011 more than 20% of patients with isolated aortic valve replacement were treated by a TAVI procedure in Germany [1]. Usually TAVI is performed using a transapical (TA) or transfemoral (TF) approach. Both approaches evolved to a highly standardized and reproducible technique. Data has been collected from multicenter registries or from single center trials [2-6]. Today, hard data from truly randomized trials comparing TAVI versus conventional AVR are still lacking. In the beginning most trials were performed using either the Edwards SAPIEN™ or the Medtronic CoreValve™. After initial enthusiasm some major concerns of the TAVI technology became obvious in larger cohort trials. Especially the incidence of paravalvular leackage, stroke and access related complications could be identified as important factors regarding the post-procedural outcome [6-8]. Driven by these concerns, the main interest of new or 2^nd^ generation devices would be the avoidance of paravalvular leakage, the reduction of sheath diameter and the repositionability of the valve prostheses. In addition the simplicity of device handling becomes more and more important to further flatten the learning curve for TAVI techniques [9-11].

## NEW TAVI DEVICES

### Edwards Lifesciences (Edwards Lifesciences Inc., California, USA)

**Fig. (1):**
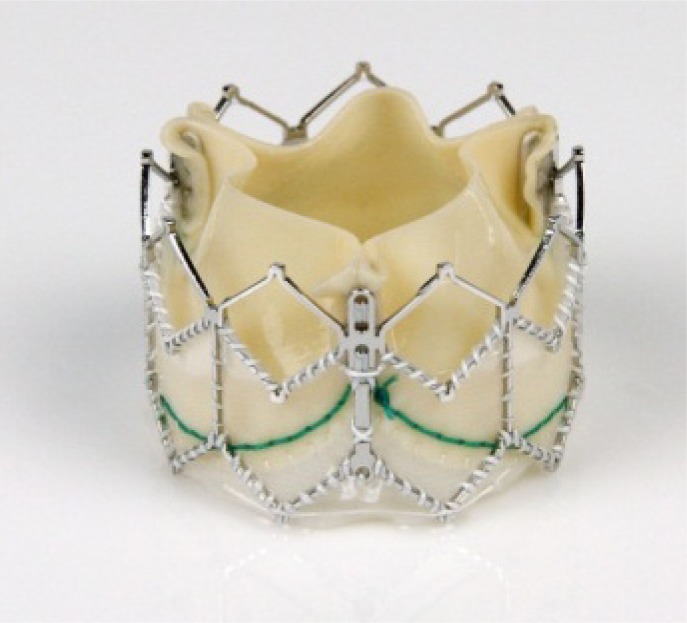
Edwards SAPIEN XT™.

The Edwards SAPIEN™ valve prosthesis was the first valve which was commercially available in Europe for TA and TF as well as in the US (currently TF only). Over the years some modifications led to the development of the most recent valve prosthesis, the SAPIEN XT™. This is a new generation of Edwards SAPIEN™, balloon expandable valve, which is already CE-mark approved and commercially available in Europe (Fig. **1**). The valve can be used for both TF and TA approaches. Compared to prior generations a cobalt-chromium frame permits thinner struts without loss of structural integrity and it provides sufficient radial force. Thinner struts and a more open design allow for a lower crimped profile and allow a smaller sheath diameter, while maintaining the valve’s radial stiffness.

**Fig. (2):**
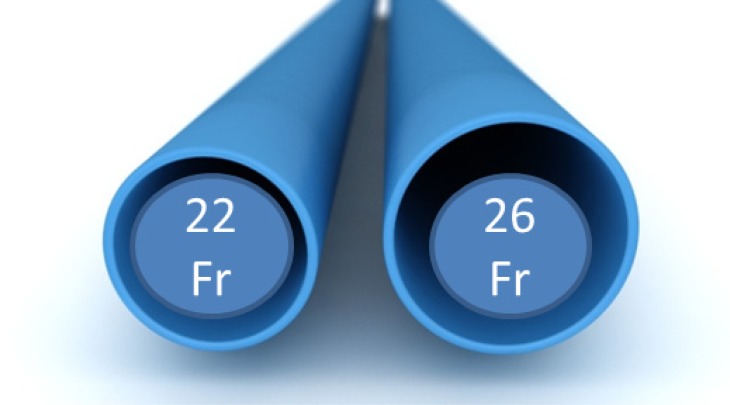
Ascendra I™(26F) introducer sheath compared to the current Ascendra II+™(22F) sheath for TA approach.

For the TA access, the delivery system improved from an initial 33F version to the current Ascendra II sheath (Fig. **2**). With 24F the diameter is significantly reduced compared to prior generations and therefore might further ease apical access (less than 1% complication rate in the multicenter PREVAIL trial) [12]. In addition, handling has been improved by incorporating a simplified de-airing button and the retrieval mechanism for the pusher has been also improved. Recently, the Ascendra+ delivery system has been introduced with a further reduction of the sheath diameter to 22F and an added “nose-cone” which will be of particular value for potential trans-aortic procedures.

**Fig. (3):**
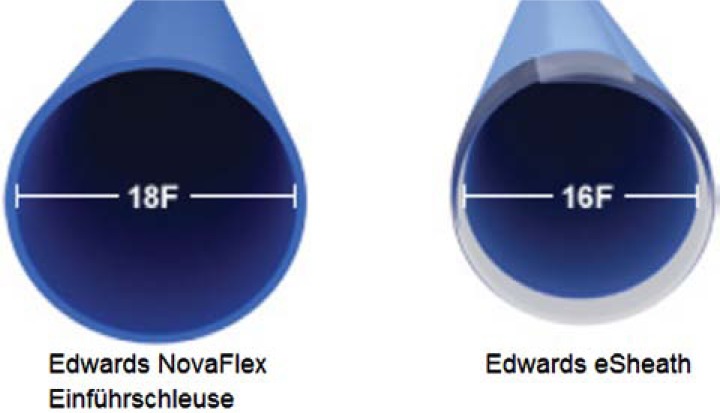
Edwards Novaflex+™ and the eSheath™ for TF approach.

For the TF access, the Novaflex+™ and the eSheath™ are used. The concept of these systems is the loading of the valve inside of the descending aorta, which allows a reduction of the sheath diameter to 16F, which is one of the major concerns regarding vascular related complications (Fig. **3**). In general, some design modifications were performed to further facilitate the handling. Available valve sizes range from 20 to 29mm and therefore allow for the treatment of patients with an aortic annulus of up to 27mm.

Further modifications, especially regarding the incidence of paravalvular leaks, supposed to be realized in the new Centera™ as well as the SAPIEN III™ valve prostheses, which recently entered “First-inMan” trials. While the SAPIEN III™ remains a ballon expandable for both TA and TF access, the Centera™ valve is a nitinol based prosthesis for TF access only, which is implanted using a motorized introducer sheath and provides the option to re-position.

### Medtronic (Medtronic, Inc., MN, USA)

**Fig. (4):**
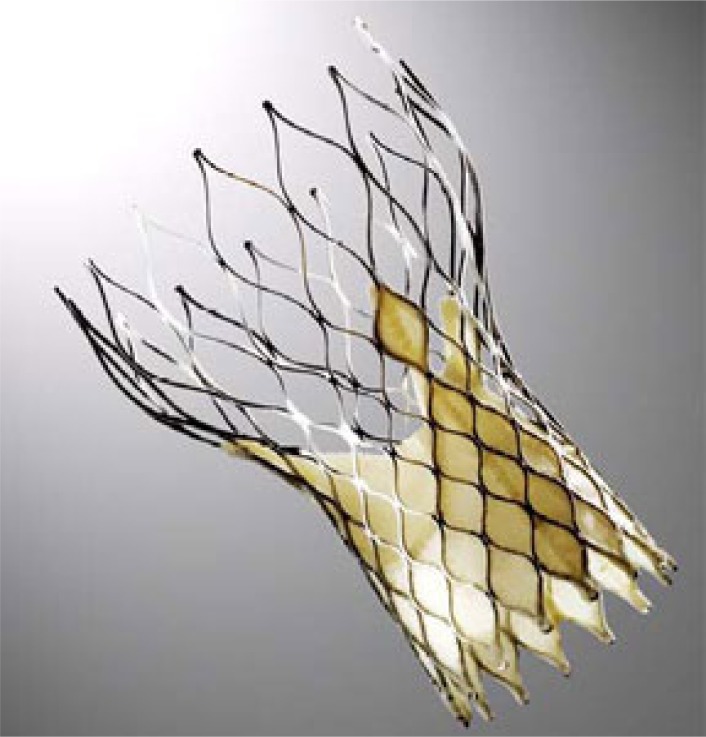
The Medtronic CoreValve™.

**Fig. (5):**
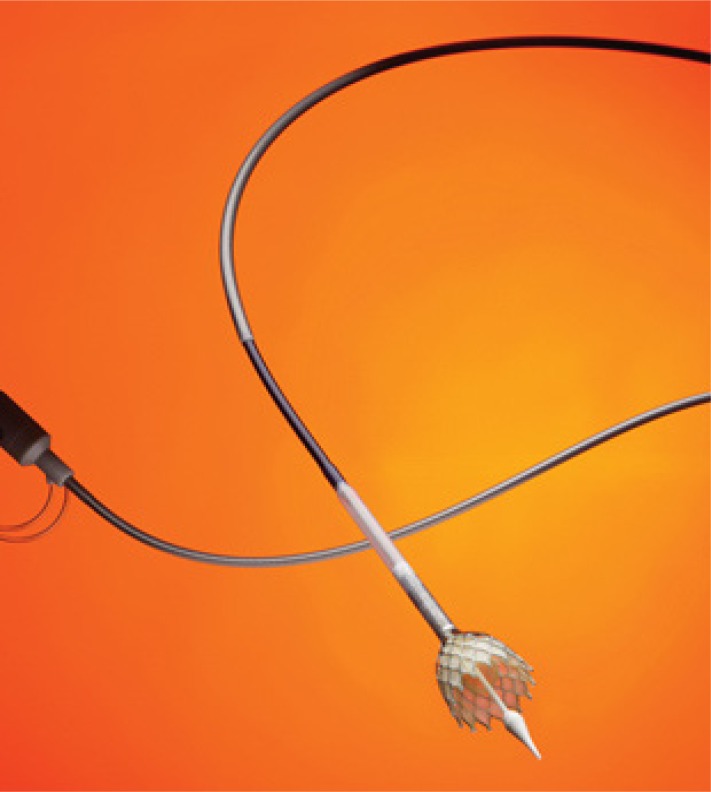
Medtronic AccuTrack™ introducer sheath (18F).

The CoreValve™ is a first generation valve for the TF approach or other retrograde access options (trans-aortic, trans-subclavian) only, which has obtained CE-mark approval in spring 2008. FDA approval for the US is expected in the end of 2012. As one of the first approved devices the prosthesis is well known. The valve consists of porcine pericardium leaflets mounted within a nitinol stent frame (Fig. **4**). Several studies have shown acceptable results for 30-day mortality as well as long-term survival [2, 3, 13]. New development of a 31mm prosthesis allows for the treatment of patients with an aortic annulus of up to 29mm. Furthermore the new 18F introducer sheath (AccuTrak™), consists of a stability layer, which allows easier insertion and a more precise implantation of the prosthesis (Fig. **5**). 

**Fig. (6):**
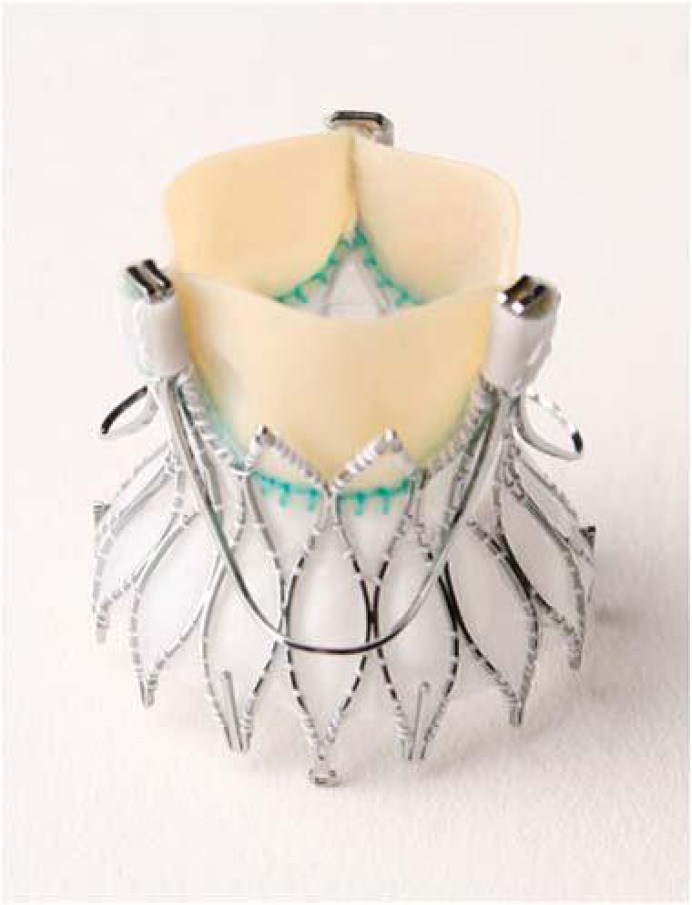
The Medtronic Engager™.

The Medtronic Engager™ Aortic Valve bioprosthesis is a second generation device originating from the initial Ventor Embracer™, which is designed for TA implantation only (Fig. **6**) [14]. The self-expanding nitinol stent consists of a main frame and a support frame, which are mounted together to form the commissural posts of the valve. The crimped valve is introduced into the aortic annulus and the three nitinol arms are then released to obtain anatomical orientation and positioning. After embracing the valvular leaflets in a correct anatomical position the valve can be totally unsheathed and implanted. The stent design results in a “predefined” position according to the individual patient anatomy. By embracing the native leaflets the theoretical risk of coronary obstruction seems to be very low. Although the valve can be partially repositioned during implantation, after total release no retrieval of the prostheses is feasible. During first clinical trials only one prosthesis size was available. Currently a redesigned version with two ? different sizes (23mm and 26mm) has entered further clinical trials. 

### Symetis (Symetis Inc, Ecublens, Switzerland)

**Fig. (7):**
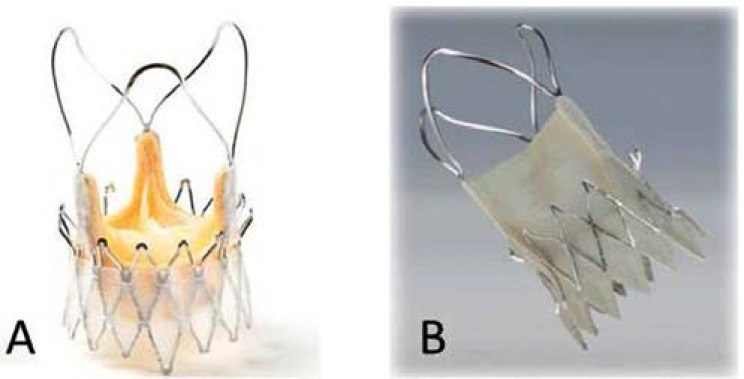
A) The Symetis Accurate™ TA. B) Symetis Accurate ™ TF.

**Fig. (8):**
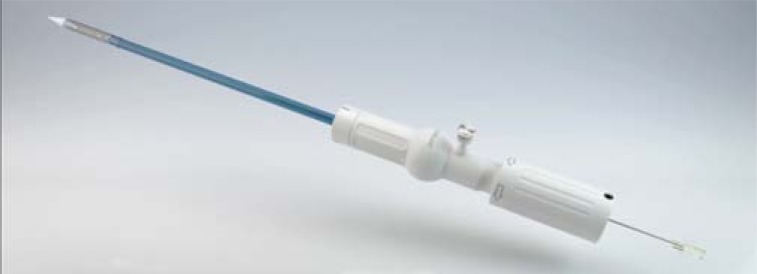
Symetis Accurate™ indtroducer sheath for TA approach.

The Symetis Acurate™ trans-catheter valve (Symetis Inc, Geneva, Switzerland), shown in (Fig. **7**), is a self-expanding nitinol stent with three stabilization arms meant to stabilize the valve in the ascending aorta, and thus prevent tilting during deployment. The device is designed to achieve an intra-annular but sub-coronary position. Inside the stent, a standard porcine tissue valve is mounted. The device design allows for anatomical rotation using enhanced imaging. The distal edge of the stent body forms the ‘upper crown’, which is not covered to minimize the risk of coronary artery obstruction. The idea of the ‘upper crown’ is to provide additional axial fixation, and, even more importantly, to facilitate and ease valve positioning with tactile feedback. The valve is retrievable until the proximal stent part is fully deployed. The prosthesis is implanted using a straight-forward 2-step implantation technique with a sheath-less delivery system that is similar in size to a 28F sheath concept (Fig. **8**). First clinical data were very promising especially regarding a low incidence of paravalvular leaks. More than 90% of patients, included in the in the pivotal trail, have shown none/trace paravalvular leack after 6 months [15, 16]. The device is available in three different sizes covering aortic annular diameters from 21 to 27 mm. CE-mark approval for Europe was granted in September 2011.

**Fig. (9):**
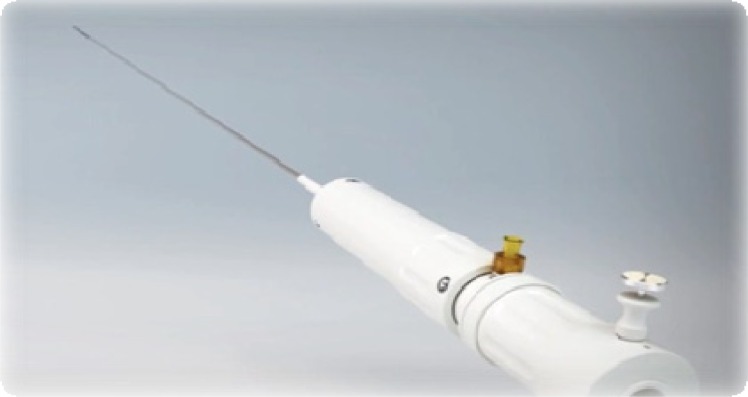
Symetis introducer sheath for TF approach.

Actually, a TF system entered a “First-in-Man” clinical trial. In contrast to the TA device, the valve consists of porcine pericardium, which allowed to reduce the diameter of the introducer sheath (Fig. **9**). Implantation and handling is similar to the TA device.

### JenaValve™ (JenaValve, Munich, Germany)

**Fig. (10):**
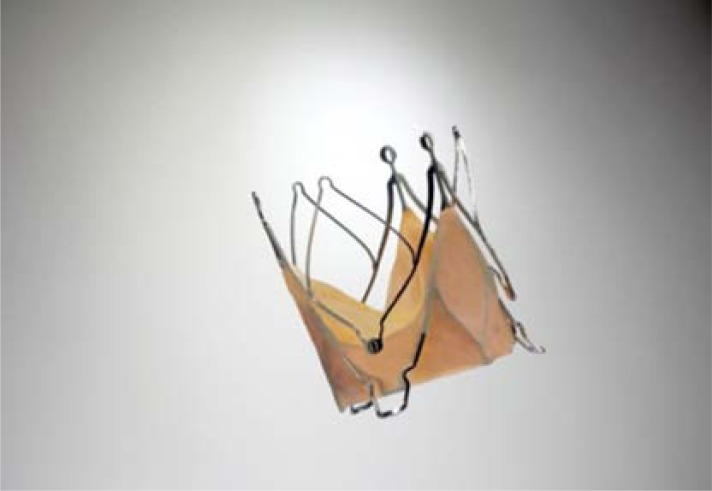
The JenaValve™.

**Fig. (11):**
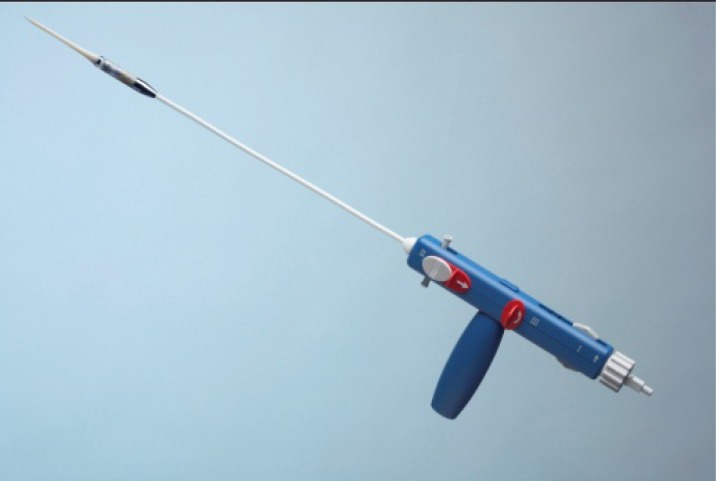
The JenaValve™ introducersheath for TA approach.

The JenaValve™ device consists of a self-expandable nitinol stent designed for subcoronary implantation using a TA approach (Fig. **10**). The device received CE-mark in September 2011 [17, 18]. Three nitinol ‘feelers’ are placed behind the native calcified aortic valve leaflets Thus, the valve is automatically implanted in an anatomically correct rotation. The stent design has a predefined implantation height and relies on axial in addition to radial fixation. A regular porcine tissue-valve is mounted inside of the stent. The valve is loaded and inserted on a sheath-less delivery system (Fig. **11**). A potential advantage of the system (and other nitinol based concepts) is that the implantation does not require rapid-pacing. Initial results have shown low incidence of moderate or severe paravalvular leckage [18]. The device is available in three different sizes allowing to treat patients with a wide range of aortic annulus diameters. Initial results are promising and a re-designed delivery system further easing the implantation technique is expected soon.

A TF version of the JenaValve is currently under development but is still in the animal trial stage.

### St. Jude Medical (St. Jude Medical, CA, USA) 

**Fig. (12):**
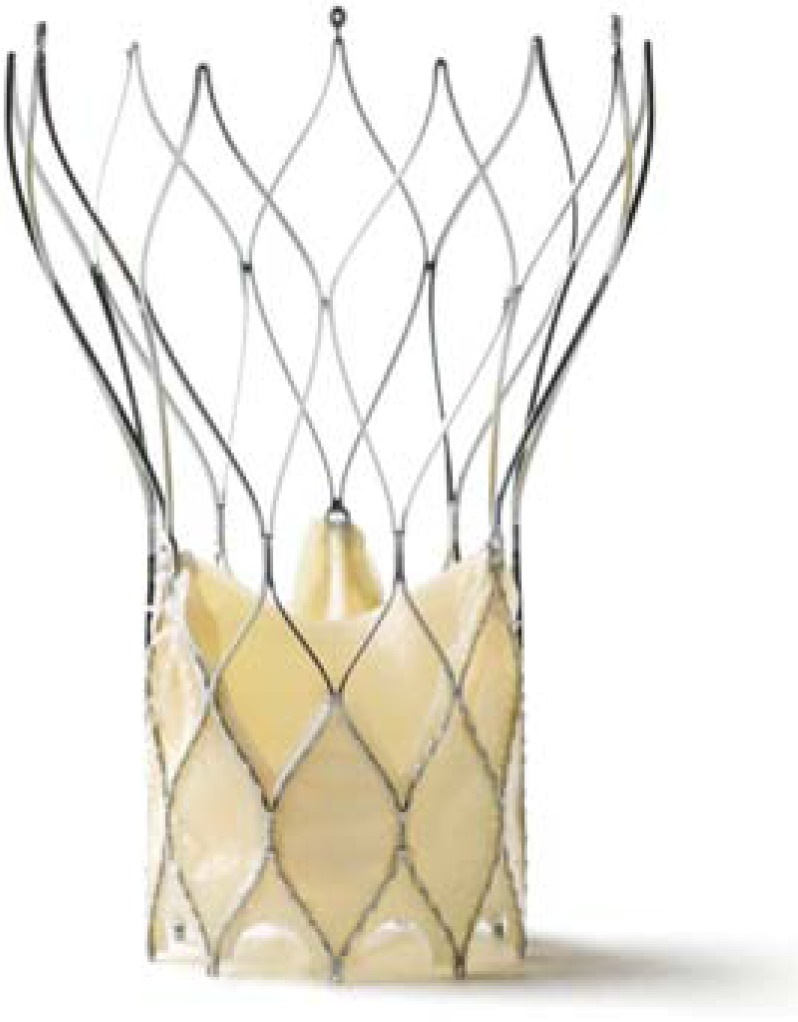
St. Jude Portico™ prosthesis.

**Fig. (13):**
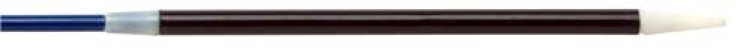
St. Jude introducer sheath for TF approach (18F).

The Portico™ prosthesis consists of bovine and porcine pericardial tissue, which is mounted on a nitinol, self-expandable stent (Fig. **12**). A large stent cell design allows access to coronaries and a low crimped profile. Low placement of leaflets within the stent frame allows for minimal protrusion into the left ventricular outflow tract meant to reduce the incidence of AV-blocks. The proximal stent part is covered by a tissue cuff designed to minimize paravalvular leaks. The prosthesis will be available in a TA and a TF version. For the TA approach the valve is delivered through a 24F introducer sheath. For the TF access the valve is implanted using an 18F introducer sheath (Fig. **13**). During implantation the valve can be re-sheathed and re-positioned or even fully retrieved prior to full deployment. Actually, a multicenter CE-mark trial for TF started in selected centers in Europe. Initiation of the pivotal trial for TA is expected soon. .

### Directflow Medical (Direct Flow Medical Inc., CA, USA)

**Fig. (14):**
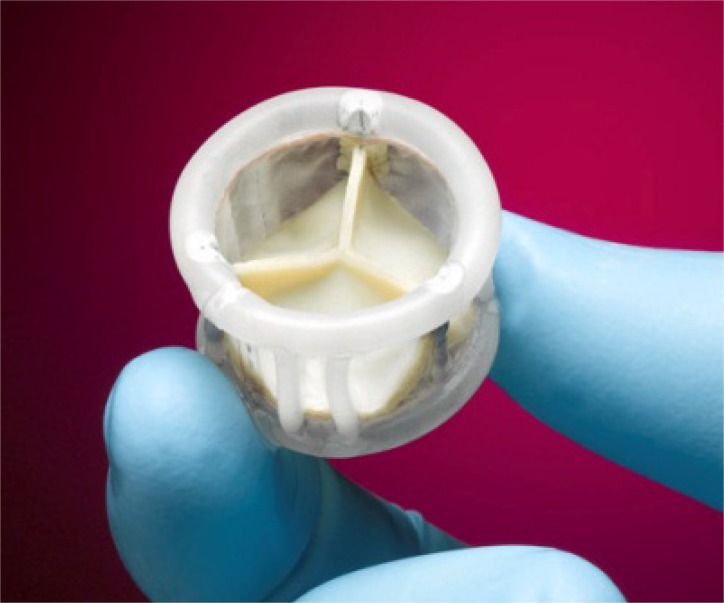
DirectFlow™.

The concept of the DirectFlow™ transfemoral transcatheter prosthesis relies on two inflatable rings for anchoring the valve at the annular level (Fig. **14**). The proximal ring is positioned just below the annulus and the distal one above. The benefit of this mechanism is that the valve is fully retrievable even after the device had been fully deployed and valve performance can be assessed. An initial feasibility trial proofed the concept and mid-term follow up is now available [19, 20]. However it also became apparent that radial forces of this design are lower than metallic based stents. Thus, in heavily calcified native aortic valves problems occurred which stimulated a re-design of the device [21]. The new version of the DirectFlow has entered clinical trials just recently.

### Boston scientific (Boston Scientific Corporation, MA, USA)

**Fig. (15):**
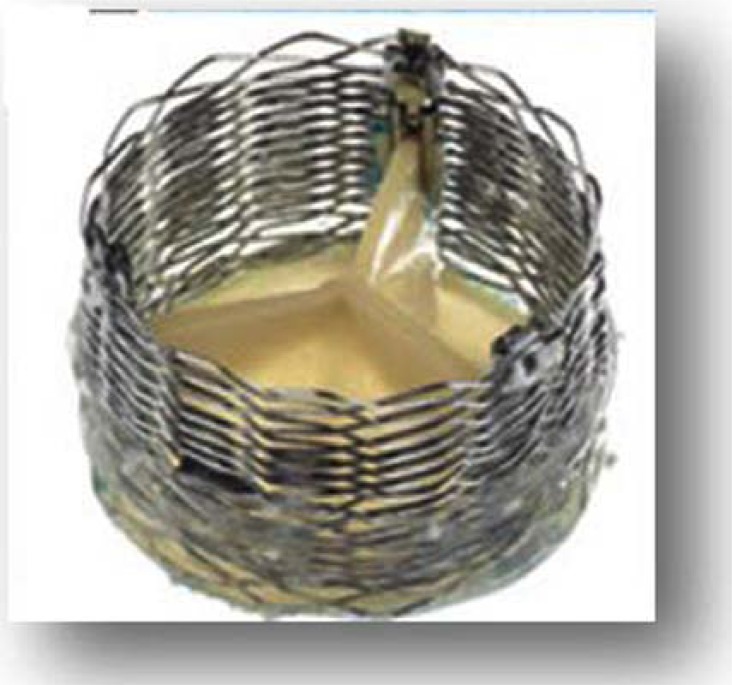
The Boston scientific Lotus™ valve.

The Lotus™ valve is based on a braided nitinol stent frame designed for a transfemoral delivery (Fig. **15**). The basic concept relies on a shortening of the nitinol cylinder which results in radial expansion providing anchoring within the aortic annulus. The key feature of the concept is that it truly allows for repositioning after valve performance assessment. In addition, an adaptive seal is meant to reduce paravalvular leak. Initial human use has been published and after further re-designing the system now has entered further clinical trials [22].

### Heart Leaflet Technologies (Heart Leaflet Technologies, Maple Grove, MN, USA)

**Fig. (16):**
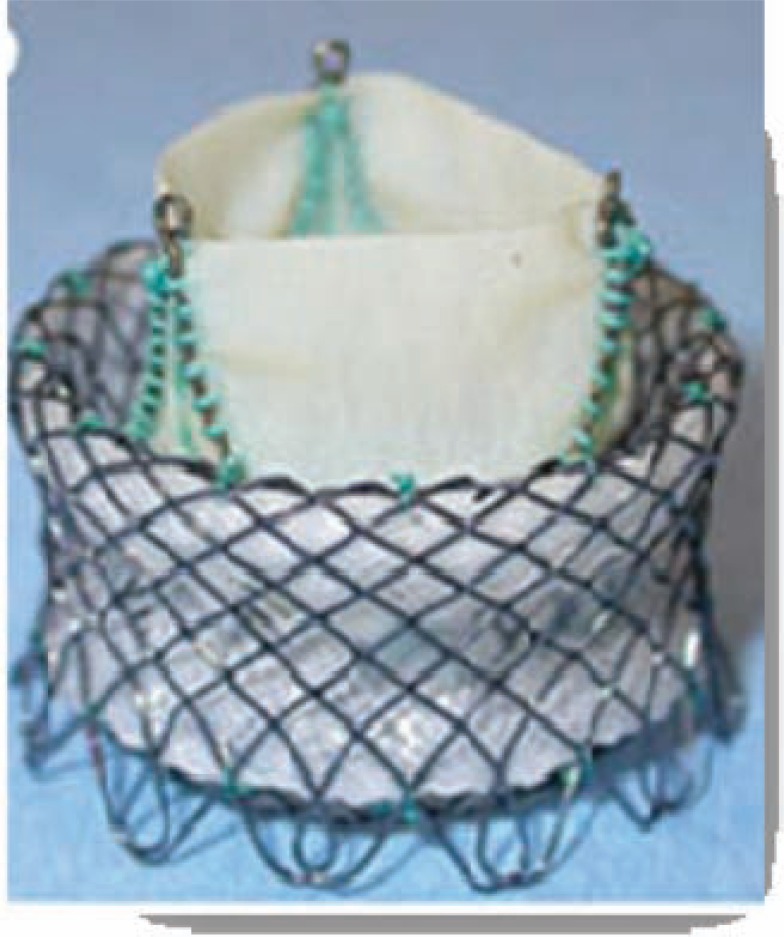
The HLT™ prosthesis.

The HLT™ transcatheter valve is based on a braided nitinol stent frame housing a tissue valve designed for retrograde (transfemoral) valve implantation (Fig. **16**). The key feature of this concept is the linear packing of the tissue valve and the nitinol stent reducing the required delivery sheath size. Prior to the actual implantation the tissue valve is “inverted” into the stent frame. First human implants have been performed and the device is currently under re-design and will enter further clinical trials shortly.

## SUMMARY

The development of TAVI is offering a truly minimally invasive option for high risk patients suffering from severe aortic stenosis. The general awareness of this new option also changed partially the referral or even more importantly the non-referral pattern in the elderly patients. Triggered by initial enthusiasm some specialists mentioned TAVI will replace conventional aortic valve replacement as the new “golden standard” procedure for patients suffering from severe aortic valve stenosis in a few years, only. At a first glimpse TAVI procedures are significantly less invasive than the conventional AVR and associated with acceptable mortality rates in high risk patients. By avoiding sternotomy, cardio-pulmonary bypass and cardiac arrest TAVI provides theoretically significant advantages and thus could be considered as an realistic alternative to conventional AVR. However, large, multicenter trials identified some major concerns related to TAVI procedures, which needs to be addressed. First of all the significant higher incidence of paravavlular leaks compared to conventional AVR is a major issue regarding the postprocedural outcome. Thus aortic regurgitation more than none/trace (>1+) after TAVI has been shown as an independent risk factor for 30-day mortality [8]. Consistently, Kodali *et al*. could demonstrate a significant influence of AR on mid-term (2-years) survival [7]. Especially patients with eccentric calcifications of the aortic leaflets are at risk for postprocedural AR and might benefit from a more liberal indication for conventional surgery allowing to resect the calcium [23]. Advanced imaging techniques are required to clearly define the severity and pattern of calcification preoperatively.

As mentioned above, new or so called “second generation” devices provide different solutions regarding these issues. However, additional leak sealing techniques, which are usually placed on the outer stent part might enlarge the size of the devices. In this context the diameter of the introducer sheath is a major limitation, especially for TF devices. Even when the sheath diameter is already significantly reduced compared to initial devices, access related complications still occur. Several trials demonstrated a significant influence of access related complications on survival. Mortality of patients with major access related complications increases significantly, especially for high surgical risk patients currently scheduled for TAVI [24-27]. In addition, leaflet thickness plays an important role regarding the sheath diameter. Therefore the use of pericardial leaflet tissue allows for further reduction of the crimped valve diameter. New ideas considering the usage of “dry” leaflets might further reduce the amount of tissue, which would allow a further significant reduction of the sheath diameter. Whether this or the aggressive crimping might have an impact on the long-term durability of the valve prostheses is unclear yet. Recently, Kiefer *et al*. could show microscopic alterations of TAVI leaflets after crimping in an experimental rat model [28]. Clinical data regarding long-term durability is lacking and due to the old high risk population currently treated hard to obtain. 

Also very important for upcoming modifications is the option of true repositionability of TAVI devices. First generation devices could not be retrieved after full deployment, which led to severe, however fortunately rare, complications, i.e. severe AR or coronary occlusion. In case of severe malposition leading to AR, implantation of a second prosthesis is the only chance to get rid of this issue except conversion to conventional surgery. Rate of a valve-in-valve implanation due to malpositioning of first prosthesis differs between 3-6% [29-31]. Some new devices can be fully retrieved until 80% of the valve stent are deployed. However, the true value of these concepts has to be awaited.

In conclusion, TAVI could already evolve to a highly standardized procedure in many centers. However, as a young technique some issues need to be considered for next generation devices to further reduce incidence of complications and mortality. Due to optimal results even for octogenarians, conventional AVR remains the “gold standard” for treatment of severe aortic stenosis, especially for younger patients. However, enthusiasm will lead to further developments and ideas to eliminate the mentioned technical shortcomings of TAVI devices, which then eventually may lead to a broadening of indications in the future.
